# Initial sirolimus dosage recommendations for pediatric patients with *PIK3CD* mutation-related immunodeficiency disease

**DOI:** 10.3389/fphar.2022.919487

**Published:** 2022-09-14

**Authors:** Xiao Chen, Jinglin Wang, Jianger Lan, Xilin Ge, Hong Xu, Yu Zhang, Zhiping Li

**Affiliations:** ^1^ Department of Pharmacy, Children’s Hospital of Fudan University, National Children’s Medical Center, Shanghai, China; ^2^ Department of Pharmacy, Union Hospital, Tongji Medical College, Huazhong University of Science and Technology, Wuhan, China; ^3^ Department of Nephrology, Children’s Hospital of Fudan University, National Children’s Medical Center, Shanghai, China

**Keywords:** sirolimus, PIK3CD mutation, immunodeficiency disease, initial dosages recommendation, pediatric patients

## Abstract

Sirolimus is used to treat pediatric patients with *PIK3CD* mutation-related immunodeficiency disease. However, the initial dosages of sirolimus remain undecided. The present study aims to explore initial dosages in pediatric patients with *PIK3CD* mutation-related immunodeficiency disease. Pediatric patients with this disease were analyzed using the population pharmacokinetic (PPK) model and the Monte Carlo simulation. Body weight and concomitant use of posaconazole were included in the final PPK model, where, under the same weight, clearances of sirolimus were 1 : 0.238 between children without and children with posaconazole. Without posaconazole, the initial dosages of sirolimus were 0.07, 0.06, 0.05, and 0.04 mg/kg/day for body weights of 10–14, 14–25, 25–50, and 50–60 kg, respectively. With posaconazole, the initial dosages of sirolimus were 0.02 mg/kg/day for body weights of 10–60 kg. This is the first attempt to build a sirolimus PPK model for recommending initial dosages in children with *PIK3CD* mutation-related immunodeficiency disease, thereby providing a reference for individualized clinical drug administration.

## Introduction

*PIK3CD* mutation-related immunodeficiency stems from autosomal dominant inheritance, leads to hyperactivity of the PI3K/Akt/mammalian target of the rapamycin (mTOR) signaling pathway, induces cell differentiation and proliferation, and results in liver, spleen, lymph node enlargement and other manifestations indicating the occurrence of disease (Burke et al., 2011; Angulo et al., 2013; Kang et al., 2020). The clinical and immunophenotypes of *PIK3CD* mutation-related immunodeficiency disease are variable, from mild to asymptomatic in adulthood, to fatal immunodeficiency in childhood, and the most common manifestations are recurrent respiratory tract infections and immune disorders (Lougaris et al., 2022). It is reported that 98% of affected children have recurrent respiratory tract infections, with pneumonia, bronchiectasis and upper respiratory tract infections being the most common (Coulter et al., 2017). The pathogens of infection in the patients include bacteria and viruses, and a small proportion has fungal infections (Brodsky and Lucas, 2021; Rivalta et al., 2021).

From an understanding of the PI3K/Akt/mTOR signaling pathway, therapeutic drugs mainly focus on the biological inhibitors of important molecules with this signaling pathway (Lucas et al., 2014). Among them, sirolimus, which directly targets mTOR and inhibits the downstream of the PI3K pathway, is currently available as one of the specific therapeutic methods used in clinical practice for children with *PIK3CD* mutation-related immunodeficiency disease (Luo et al., 2018; Kang et al., 2020; Singh et al., 2020; Redenbaugh and Coulter, 2021; Rivalta et al., 2021). However, appropriate initial dosages of sirolimus in pediatric patients with this disease remain uncertain. In addition, a narrow therapeutic range and considerable inter- and intra-individual pharmacokinetic variabilities make it hard to develop a regimen of sirolimus initial dosages (Wang et al., 2020; Chen et al., 2021b; Zhang et al., 2022). There is thus an urgent need to provide an accurate and individualized sirolimus administration regimen for the treatment of pediatric patients with *PIK3CD* mutation-related immunodeficiency disease.

In clinical practice, therapeutic drug monitoring (TDM) is often used to detect sirolimus concentration in order to adjust the dosage to reach the required range of treatment window. The next sirolimus dosage is often optimized in light of the drug concentration level to achieve the purpose of synergism and toxicity reduction. However, this dosage adjustment needs to be based on a measurement of existing sirolimus concentration, and so this method is not useful for determining initial dosages as no sirolimus levels are available for reference. Population pharmacokinetic (PPK), combined with a Monte Carlo simulation, provide feasible technical support for solving initial dosage recommendations in clinical practice. In recent years, more and more related research has been reported, which further verifies the reliability and practicability of this method (Jaruratanasirikul et al., 2019; Ren et al., 2019; Ghoneim et al., 2021; Rinaldi et al., 2021; Tang Girdwood et al., 2022). Hence, the present study recommends initial dosages of sirolimus in pediatric patients with *PIK3CD* mutation-related immunodeficiency disease based on PPK and a Monte Carlo simulation.

## Methods

### Patients

Pediatric patients with *PIK3CD* mutation-related immunodeficiency disease at the Children’s Hospital of Fudan University (Shanghai, China) between September 2017 and January 2022 were retrospectively collected. Inclusion criteria were as follows: 1) treated with sirolimus, and 2) carrying out TDM for sirolimus. The trough level of sirolimus concentration was tested using the Emit 2000 Sirolimus Assay (Siemens Healthcare Diagnostics Inc.). The study was approved by the Research Ethics Committee of the Children’s Hospital of Fudan University (Ethical code: [2019] 019). As the study was retrospective, it was approved by the ethics committee of our hospital without the need for written informed consent.

### Model establishment

A first-order conditional estimation method with interaction (FOCE-I method) by NONMEM (edition 7, ICON Development Solutions, Ellicott City, MD, USA) was used to build up the PPK model, in which the pharmacokinetic parameters included apparent oral clearance (CL/F), volume of distribution (V/F), and absorption rate constant (Ka, fixed at 0.485/h (Wang et al., 2020)).

Eq. 1 shows the inter-individual variability:

$${\text{A}}_{\text{i}}=\text{TV}\left(\text{A}\right)\text{×exp}\left(\eta_{\text{i}}\right)$$ (1)

where A_i_ represents the individual parameter value; TV(A) represents the typical individual parameter value; and η_i_ represents symmetrical distribution, which is a random term with zero mean and variance omega^2^ (ω^2^).

Equations 2-4 show the residual unexplained variability (RUV), Eq. 2 is described with an additive model, Eq. 3 is described with a proportional model, and Eq. 4 is described with a mixed model:

$${\text{B}}_{\text{i}}{=\text{C}}_{\text{i}}+\varepsilon_{1}$$ (2)

$${\text{B}}_{\text{i}}{=\text{C}}_{\text{i}}\text{∗}\left(1+\varepsilon_{1}\right)$$ (3)

$${\text{B}}_{\text{i}}{=\text{C}}_{\text{i}}\text{∗}\left(1+\varepsilon_{1}\right)+\varepsilon_{2}$$ (4)

where B_i_ represents the observed concentration; C_i_ represents the individual predicted concentration; and ε_1_, ε_2_ represents symmetrical distribution, which is a random term with zero mean and variance sigma^2 (σ^2^).

Eq. 5 shows the relation of the pharmacokinetic parameters to weight:

$${\text{D}}_{\text{i}}{=\text{D}}_{\text{std}}\text{×}{\left({\text{E}}_{\text{i}}/{\text{E}}_{\text{std}}\right)}^{\text{index}}$$ (5)

where D_i_ represents the *i*th individual parameter; E_i_ represents the *i*th individual weight; E_std_ represents the standard weight of 70 kg; D_std_ represents the typical individual parameter, whose weight is E_std_; and index represents the allometric coefficient: 0.75 for the CL/F and 1 for the V/F (Anderson and Holford, 2008).

Eqs 6, 7 show the pharmacokinetic parameters between continuous covariates or categorical covariates, respectively:

$${\text{F}}_{\text{i}}=\text{TV}\left(\text{F}\right)\text{×}{\left({\text{Cov}}_{\text{i}}/{\text{Cov}}_{\text{m}}\right)}^{\theta }$$ (6)

$${\text{F}}_{\text{i}}=\text{TV}\left(\text{F}\right)\text{×}\left(1+\theta \text{×}{\text{Cov}}_{\text{i}}\right)$$ (7)

where F_i_ represents the individual parameter value; TV(F) represents the typical individual parameter value; θ represents the parameter to be estimated; Cov_i_ represents the covariate of the *i*th individual; and Cov_m_ represents the population median for the covariate. Potential covariates in the analysis include gender, age, albumin, alanine transaminase, aspartate transaminase, creatinine, urea, total protein, total bile acid, direct bilirubin, total bilirubin, hematocrit, hemoglobin, mean corpuscular hemoglobin, mean corpuscular hemoglobin concentration, and co-medications cefdinir, omeprazole, posaconazole, and sulfamethoxazole. Objective function value (OFV) changes are used as covariate inclusion criteria, where the decrease of OFV greater than 3.84 (*p* < 0.05) is considered sufficient for inclusion in the base model, and the increase of OFV greater than 6.63 (*p* < 0.01) is considered sufficient for significance in the final model (Chen et al., 2021b).

### Model evaluation

The model visualization was evaluated using goodness-of-fit plots (conditional weighted residuals (WRES) *vs.* time, observation *vs.* population prediction, observation *vs.* individual prediction, conditional WRES *vs.* population prediction, and conditional WRES *vs.* individual prediction). The model distribution was evaluated by distribution of weighted residuals for model (density *vs.* weighted residuals, quantities of weighted residuals *vs.* quantities of normal). In addition, the medians and 2.5th-97.5th percentiles of the results from bootstraps (*n* = 1,000) were compared with final model parameters.

### Model simulation

The Monte Carlo method was used to simulate initial dosages of sirolimus in pediatric patients with *PIK3CD* mutation-related immunodeficiency disease, based on the final model, and where the target concentration window of sirolimus was 5–15 ng/ml. In the present study, we found weight and concomitant medication of posaconazole affected sirolimus clearance. Therefore, we simulated two different situations based on whether the children were co-administered with posaconazole or not. In each situation, 1,000 virtual pediatric patients with *PIK3CD* mutation-related immunodeficiency disease were simulated in ten dosages (0.01, 0.02, 0.03, 0.04, 0.05, 0.06, 0.07, 0.08, 0.09, 0.10 mg/kg/day) for six weight groups (10, 20, 30, 40, 50, 60 kg), respectively. The probabilities of achieving the target concentration window were used as the evaluation criteria, and the probabilities of exceeding the upper limit of the treatment window (15 ng/ml) at 1,000 simulated concentration were used as an evaluation of safety.

## Results

### Patients

A total of 24 pediatric patients with *PIK3CD* mutation related-immunodeficiency disease were included, 16 boys and 8 girls, whose ages were from 2.15–17.92 years and weight from 10–63 kg. The patients having oral sirolimus were receiving it once daily. Demographic data of the pediatric patients with *PIK3CD* mutation-related immunodeficiency disease and drug combinations are shown in Table 1.

**TABLE 1:** Demographic data of patients and drug combination (*n* = 24).

	Mean ± SD	Median (range)
Gender (boys/girls)	16/8	—
Age (years)	8.79 ± 3.65	7.94 (2.15–17.92)
Weight (kg)	23.46 ± 7.72	22.00 (10.00–63.00)
Albumin (g/L)	40.17 ± 5.52	40.90 (26.10–50.20)
Alanine transaminase (IU/L)	15.71 ± 6.16	15.90 (2.82–28.60)
Aspartate transaminase (IU/L)	28.31 ± 8.95	25.96 (15.20–51.50)
Creatinine (μmol/L)	31.06 ± 10.78	28.00 (17.00–58.00)
Urea (mmol/L)	3.82 ± 0.98	3.57 (2.60–6.70)
Total protein (g/L)	70.67 ± 7.36	70.80 (55.80–87.30)
Total bile acid (μmol/L)	6.35 ± 4.12	5.80 (0.90–13.70)
Direct bilirubin (μmol/L)	2.79 ± 1.42	2.40 (0.80–6.30)
Total bilirubin (μmol/L)	8.79 ± 3.89	9.25 (2.90–16.60)
Hematocrit (%)	35.96 ± 6.80	35.01 (24.20–50.50)
Hemoglobin (g/L)	116.67 ± 24.72	119.50 (71.00–166.00)
Mean corpuscular hemoglobin (pg)	25.77 ± 2.80	26.85 (18.70–28.60)
Mean corpuscular hemoglobin concentration (g/L)	323.58 ± 24.58	329.00 (273.00–364.00)
Number of co-medications		
Cefdinir	11	—
Omeprazole	4	—
Posaconazole	2	—
Sulfamethoxazole	12	—

### Modeling

The RUV model with additive error method was selected. Eqs 8, 9 show the final model:

$$\text{CL}/\text{F}=10\text{.}4\text{×}{\left(\text{WT}/70\right)}^{\text{0}\text{.}75}\text{×}\left(1-\text{0}\text{.}762\text{×POS}\right)$$ (8)

V/F=583×(WT/70) (9)

where WT represents body weight, and POS represents posaconazole. Where a patient was co-administered posaconazole, the POS value is 1, otherwise the POS value is 0.

### Evaluation

**FIGURE 1:**
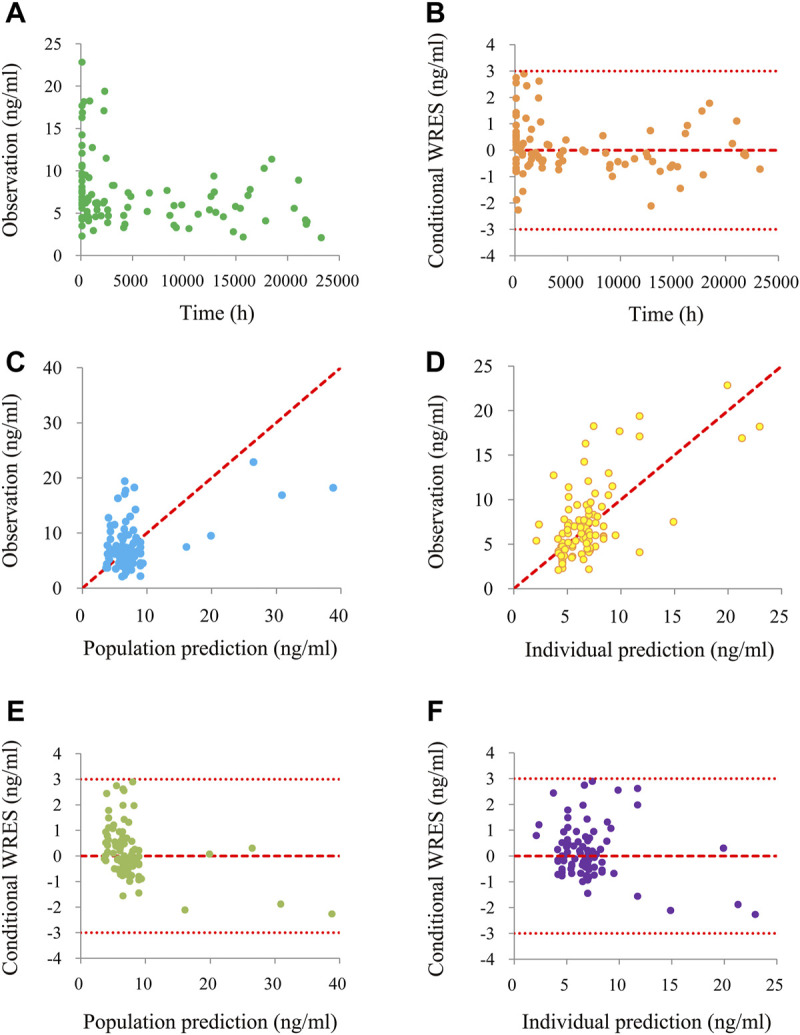
The goodness-of-fit plots of model. **(A)** Time-concentration diagram. **(B)** Conditional weighted residuals (WRES) *vs.* time. **(C)** Observation *vs.* population prediction. **(D)** Observation *vs.* individual prediction. **(E)** Conditional WRES *vs.* population prediction. **(F)** Conditional WRES *vs.* individual prediction.

**FIGURE 2:**
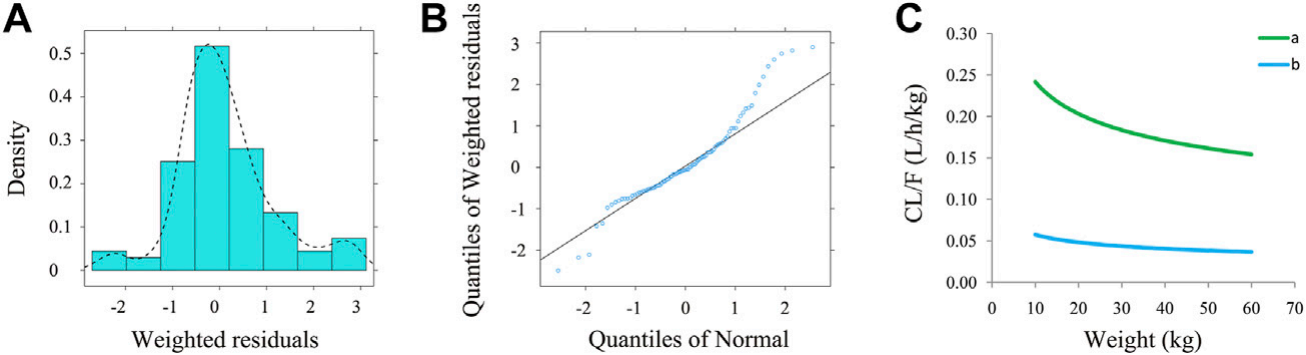
The evaluation of normality and sirolimus clearance rate. **(A)** Density *vs.* weighted residuals. **(B)** Quantities of weighted residuals *vs.* quantities of normal. **(C)** Sirolimus clearance rate: **(a)** children with *PIK3CD* mutation-related immunodeficiency disease without posaconazole, and **(b)** children with *PIK3CD* mutation-related immunodeficiency disease with posaconazole.

Figure 1 is a time-concentration diagram and model evaluation, where Figure 1A is observation *vs.* time, and Figures 1B–F are goodness-of-fit plots, showing good model fitting effect. Figures 2A,B are the distribution of weighted residuals that follow the normal distribution. In addition, Figure 2C is the sirolimus clearance rate, and under the same weight, the clearances of sirolimus were 1 : 0.238 between children without posaconazole and children with posaconazole. Table 2 are bootstraps, and the parameter estimates of the final model were within a 95% confidence interval of 1,000 bootstraps, showing that the model is reliable.

**TABLE 2:** Parameter estimates of final model and bootstrap validation.

Parameter	Estimate	SE	Bootstrap		Bias (%)
			Median	95% confidence interval	
CL/F (L/h)	10.4	0.173	9.43	[1.42, 12.10]	−9.33
V/F (L)	583	0.465	484	[11, 783]	−16.98
Ka (h^−1^)	0.485 (fixed)	--	--	--	--
θ_POS_ on CL/F	-0.762	1.144	-0.706	[-0.887, -0.214]	−7.35
ω_CL/F_	0.303	2.208	0.200	[0.033, 0.509]	−33.99
σ_1_	3.674	0.419	3.450	[2.324, 4.525]	−6.10

95% confidential interval was displayed as the 2.5th and 97.5th percentile of bootstrap estimates. CL/F, apparent oral clearance (L/h); V/F, apparent volume of distribution (L); Ka, absorption rate constant (h^−1^); θ_POS_, on CL/F was the coefficient of posaconazole; ω_CL/F_, inter-individual variability of CL/F; σ_1_, residual variability, additive error; Bias, prediction error, Bias = (Median-Estimate)/Estimate×100%.

### Simulation

**FIGURE 3:**
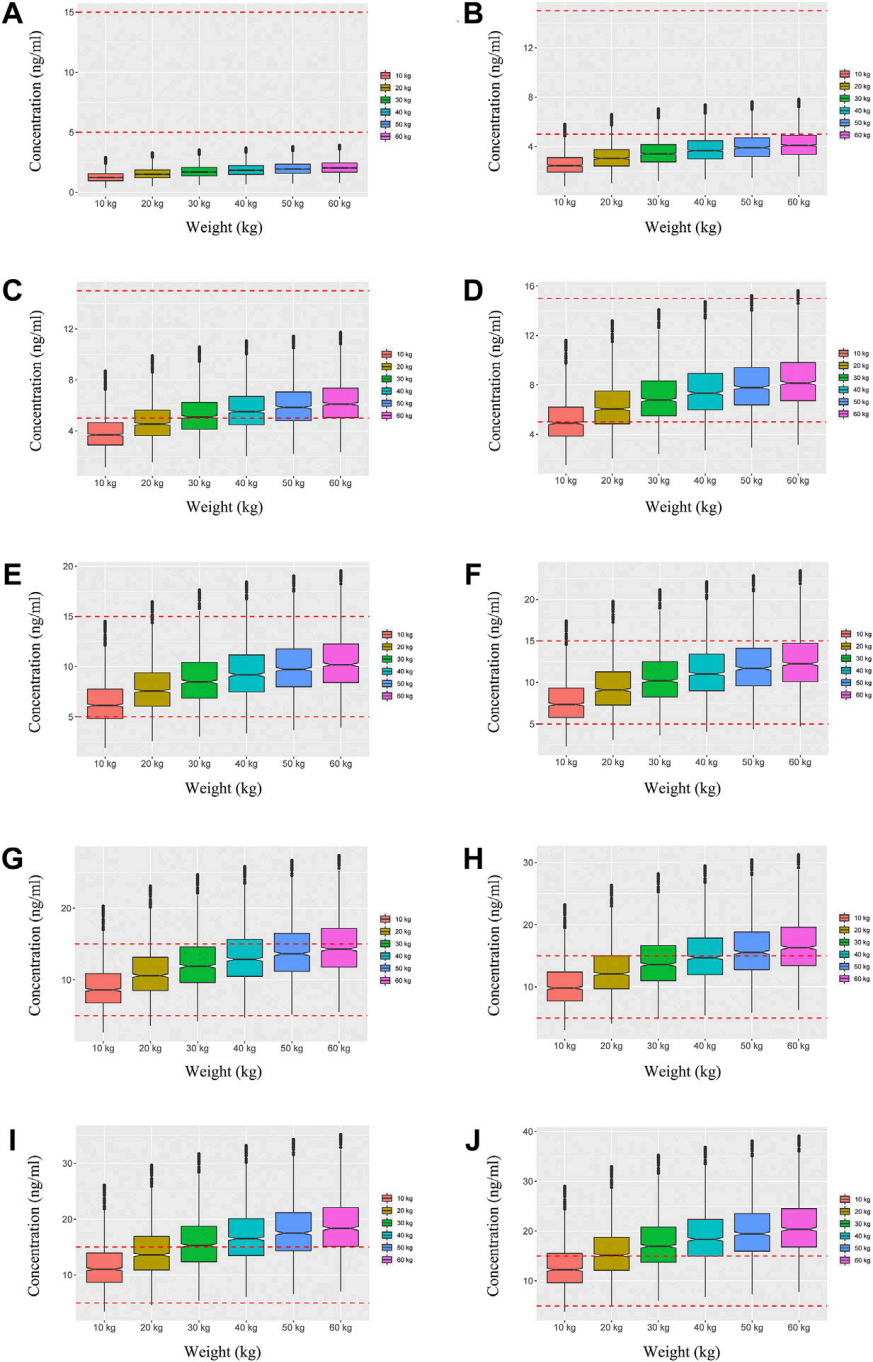
Simulation of sirolimus concentrations in children with *PIK3CD* mutation-related immunodeficiency disease without posaconazole. **(A)** 0.01 mg/kg/day sirolimus dosage; **(B)** 0.02 mg/kg/day sirolimus dosage; **(C)** 0.03 mg/kg/day sirolimus dosage; **(D)** 0.04 mg/kg/day sirolimus dosage; **(E)** 0.05 mg/kg/day sirolimus dosage; **(F)** 0.06 mg/kg/day sirolimus dosage; **(G)** 0.07 mg/kg/day sirolimus dosage; **(H)** 0.08 mg/kg/day sirolimus dosage; **(I)** 0.09 mg/kg/day sirolimus dosage; **(J)** 0.10 mg/kg/day sirolimus dosage.

**FIGURE 4:**
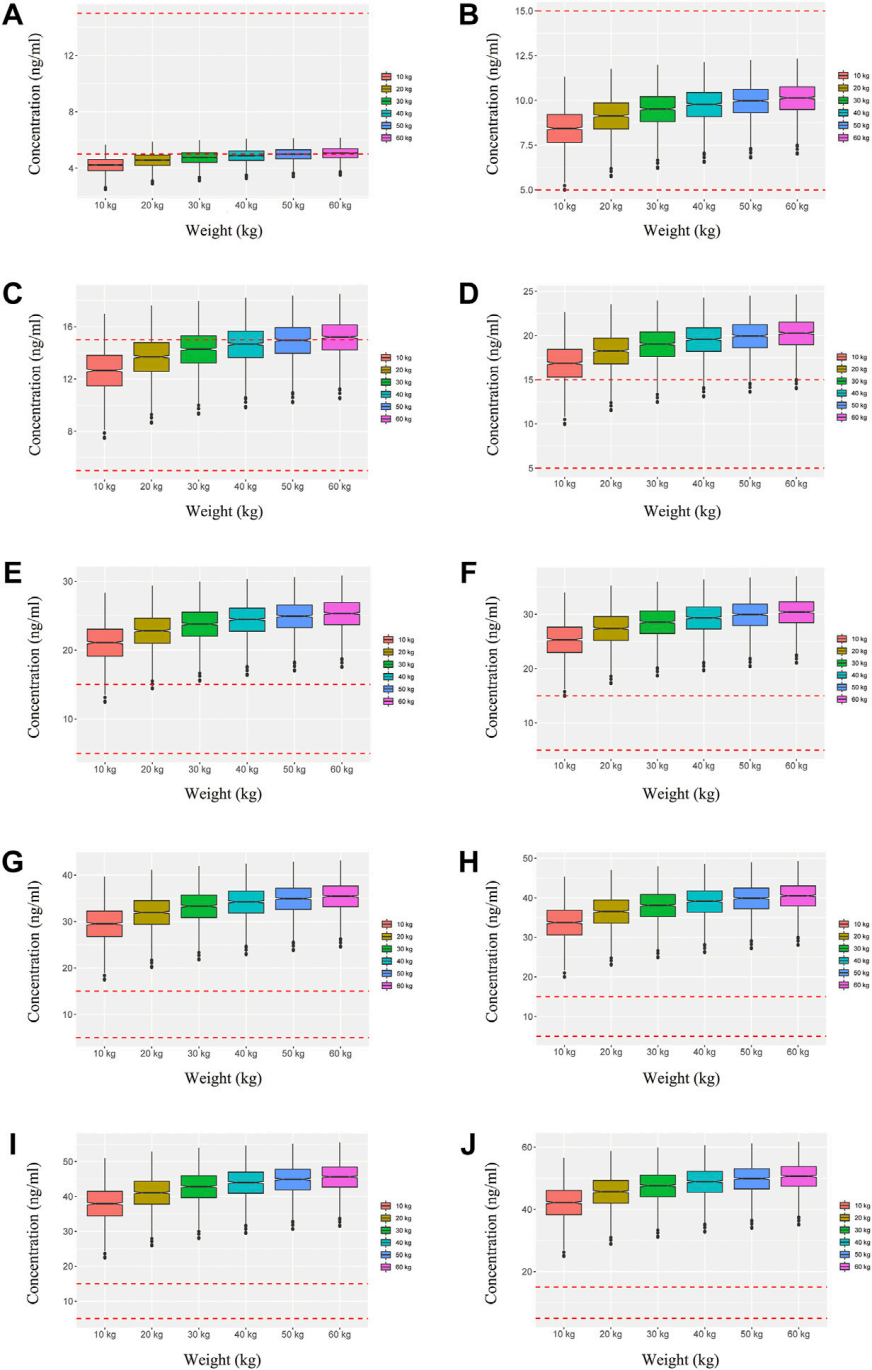
Simulation of sirolimus concentrations in children with *PIK3CD* mutation-related immunodeficiency disease with posaconazole. **(A)** 0.01 mg/kg/day sirolimus dosage; **(B)** 0.02 mg/kg/day sirolimus dosage; **(C)** 0.03 mg/kg/day sirolimus dosage; **(D)** 0.04 mg/kg/day sirolimus dosage; **(E)** 0.05 mg/kg/day sirolimus dosage; **(F)** 0.06 mg/kg/day sirolimus dosage; **(G)** 0.07 mg/kg/day sirolimus dosage; **(H)** 0.08 mg/kg/day sirolimus dosage; **(I)** 0.09 mg/kg/day sirolimus dosage; **(J)** 0.10 mg/kg/day sirolimus dosage.

**FIGURE 5:**
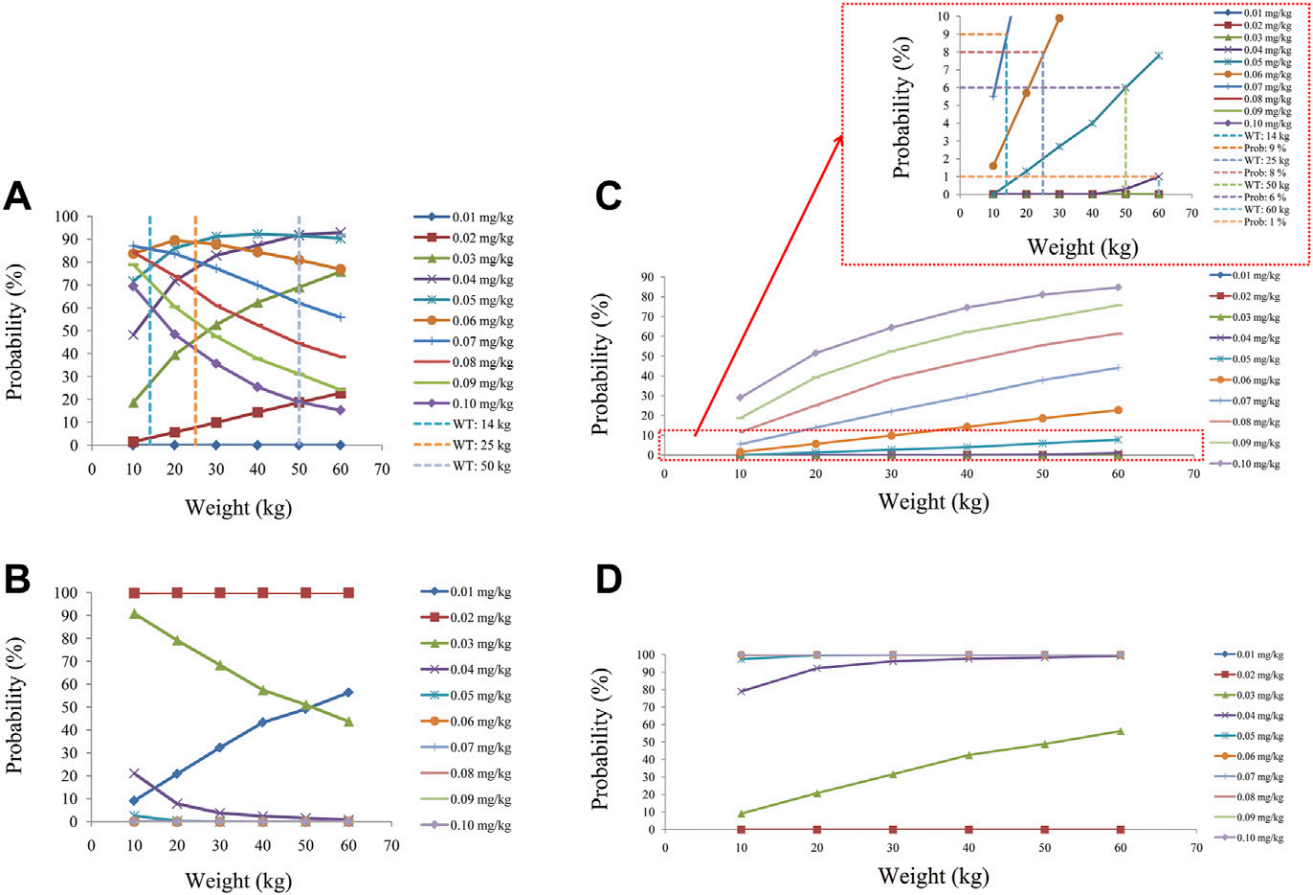
Evaluation of effectiveness and safety. **(A)** Probability of achieving target concentrations from children with *PIK3CD* mutation-related immunodeficiency disease without posaconazole. **(B)** Probability of achieving target concentrations from children with *PIK3CD* mutation-related immunodeficiency disease with posaconazole. **(C)** Probability of exceeding the upper limit of target concentrations from children with *PIK3CD* mutation-related immunodeficiency disease without posaconazole. **(D)** Probability of exceeding the upper limit of the target concentrations from children with *PIK3CD* mutation-related immunodeficiency disease with posaconazole.

We simulated two different situations, based on whether the children were co-administered posaconazole or not. Figure 3 is children with *PIK3CD* mutation-related immunodeficiency disease without posaconazole, and Figures 3A–J are ten dosages (0.01, 0.02, 0.03, 0.04, 0.05, 0.06, 0.07, 0.08, 0.09, 0.10 mg/kg/day). Figure 4 is children with *PIK3CD* mutation-related immunodeficiency disease with posaconazole, and Figures 4A–J are ten dosages (0.01, 0.02, 0.03, 0.04, 0.05, 0.06, 0.07, 0.08, 0.09, 0.10 mg/kg/day). The two red dashed lines represent the range of sirolimus concentrations. Further analysis revealed that without posaconazole, the initial dosages of sirolimus were 0.07, 0.06, 0.05, and 0.04 mg/kg/day for body weights of 10–14, 14–25, 25–50, and 50–60 kg, respectively, which is shown in Figure 5A. With posaconazole, the initial dosages of sirolimus were 0.02 mg/kg/day for body weights of 10–60 kg, as shown in Figure 5B. In addition, the safety of the recommended dosages was also considered in this study. As shown in Figure 5C, for pediatric patients with *PIK3CD* mutation-related immunodeficiency disease without posaconazole, the probabilities of exceeding the upper limit of the target concentration were less than 9%, 8%, 6%, and 1%, for the dosages of sirolimus 0.07, 0.06, 0.05, and 0.04 mg/kg/day, respectively. As shown in Figure 5D, for pediatric patients with *PIK3CD* mutation-related immunodeficiency disease with posaconazole, the concentrations from dosages of 0.02 mg/kg/day sirolimus were all within the treatment window, and there was no value exceeding the upper limit of the target concentration. Table 3 is initial dosage recommendation of sirolimus.

**TABLE 3:** Initial dosage recommendation of sirolimus.

Without posaconazole		With posaconazole	
Body weight (kg)	Dose (mg/kg/day)	Body weight (kg)	Dose (mg/kg/day)
10–14	0.07	10–60	0.02
14–25	0.06	—	—
25–50	0.05	—	—
50–60	0.04	—	—

## Discussion

*PIK3CD* mutation-related immunodeficiency disease, which is also known as activated phosphoinositide 3-kinase δ syndrome (APDS)-1, and which is mainly due to an overactivated PI3K/Akt/mTOR signaling pathway, can be treated from the cause using sirolimus, an mTOR inhibitor (Luo et al., 2018; Kang et al., 2020; Singh et al., 2020; Redenbaugh and Coulter, 2021; Rivalta et al., 2021). However, the significant inter-individual variability and a narrow therapeutic index of sirolimus make it difficult to set the dosages for administration (Chen et al., 2021b; Zhang et al., 2022). The present study aims to explore the initial dosage of sirolimus in children with *PIK3CD* mutation-related immunodeficiency disease.

In actual practice, conditional WRES is usually with FOCE-I, and of course this is acceptable. Thus, conditional WRES are all with FOCE-I in the following: Jing et al.’s ( 2021) pharmacokinetic analysis and dosing guidelines for tacrolimus co-administration with wuzhi capsules in Chinese renal transplant recipients; Li et al.’s (2021) study of dose tailoring of tacrolimus, based on a non-linear pharmacokinetic model in children with refractory nephrotic syndrome; Yu et al.’s (2021) study of pharmacokinetics and optimization of polymyxin B dosing in adult patients with various renal functions; Ma et al.’s (2020) study of the pharmacokinetics of vancomycin in kidney transplant recipients; and Cai et al.’s (2020)model building and parameter optimization in pharmacokinetics and dosing regimen optimization of tacrolimus in Chinese lung transplant recipients.

In the final PPK model of sirolimus from pediatric patients with *PIK3CD* mutation-related immunodeficiency disease, body weight and concomitant use of posaconazole were included, where, under the same weight; the clearances of sirolimus were 1 : 0.238 between children without posaconazole and children with posaconazole. In other words, antifungal therapy with posaconazole, the second generation of triazole antifungal agent, significantly reduced clearance of sirolimus in children with *PIK3CD* mutation-related immunodeficiency disease, because posaconazole is an inhibitor of the cytochrome P450 (CYP) isoenzyme CYP3A4, and sirolimus is a substrate of the enzyme (Moton et al., 2009). Previously, co-administration of posaconazole and sirolimus were reported clinically, mainly focused on hematopoietic stem cell transplant recipients (Kubiak et al., 2012; Cho et al., 2015; Greco et al., 2016), and liver transplant recipients (Dahlan et al., 2012; Deyo et al., 2017). However, the drug interaction between posaconazole and sirolimus in children with *PIK3CD* mutation-related immunodeficiency disease has not been reported. The present study is the first exploring this particular group of pediatric patients with *PIK3CD* mutation-related immunodeficiency disease.

Further, the Monte Carlo method was used to simulate initial dosages of sirolimus in pediatric patients with *PIK3CD* mutation-related immunodeficiency disease based on the final model, and where the target concentration window of sirolimus was 5–15 ng/ml. Of course, there were differences in the range of sirolimus requirement for different diseases. For example, in kaposiform hemangioendothelioma patients, the target concentration of sirolimus was 10–15 ng/ml (Ji et al., 2022). In relapsed/refractory autoimmune cytopenias patients, all of the patients achieved a goal trough level, ranging between 4.6 and 20 ng/ml by the first measurement (Bride et al., 2016). In chronic immune thrombocytopenia patients, the sirolimus range was 5–15 ng/ml (Mousavi-Hasanzadeh et al., 2020). In tuberous sclerosis complex patients, the sirolimus range was 5–15 ng/ml (Wang et al., 2020). In pediatric patients with lymphangioma, the sirolimus range was also 5–15 ng/ml (Chen et al., 2021a). We simulated two different situations based on whether the children were co-administered posaconazole or not. Without posaconazole, the initial dosages of sirolimus were 0.07, 0.06, 0.05, and 0.04 mg/kg/day for body weights of 10–14, 14–25, 25–50, and 50–60 kg in pediatric patients with *PIK3CD* mutation-related immunodeficiency disease, respectively. With posaconazole, the initial dosages of sirolimus were 0.02 mg/kg/day for body weights of 10–60 kg. In addition, the safety of the recommended dosages was also considered in this study. For pediatric patients with *PIK3CD* mutation-related immunodeficiency disease without posaconazole, the probabilities of exceeding the upper limit of the target concentration were less than 9%, 8%, 6%, and 1%, for the dosages of sirolimus, 0.07, 0.06, 0.05, and 0.04 mg/kg/day respectively. For pediatric patients with *PIK3CD* mutation-related immunodeficiency disease with posaconazole, the concentrations from dosages of 0.02 mg/kg/day sirolimus were all within the treatment window, and there was no value exceeding the upper limit of the target concentration. Based on these data, we may determine that the current dosing regimens are safe and meet clinical requirements.

Additionally, in relation to other diseases, Mousavi-Hasanzadeh et al. (2020) explored the dosages of sirolimus for the treatment of pediatric chronic immune thrombocytopenia in depth. They found the sirolimus loading dose of 6 mg/m^2^ body surface area >40 kg, and 3 mg/m^2^ body surface area <40 kg, and a maintenance dose of 2 mg/m^2^ body surface area >40 kg, and 1 mg/m^2^ body surface area <40 kg daily, could be effective in treating chronic immune thrombocytopenia in children. Meanwhile, as the limiting condition of narrow therapeutic border and variable bioavailability, sirolimus blood levels were monitored, whose reference range was 5–15 ng/ml (Mousavi-Hasanzadeh et al., 2020). Although the sirolimus dosing protocol in that study is not completely consistent with the present study, the guiding idea for both was to achieve drug administration guided by drug exposure based on TDM. Of course, this is the recognized optimization basis for sirolimus administration in the treatment of different diseases.

However, this study also had some limitations. Overall, due to the lower morbidity of *PIK3CD* mutation-related immunodeficiency disease, the number of pediatric patients included in the present study is small. In addition, the retrospective study design may influence the target concentrations of sirolimus to some extent. It will therefore be necessary to expand the number of pediatric patients included in future prospective studies to further verify our conclusions.

## Conclusion

This is the first effort to build a sirolimus PPK model and recommend the initial dosages in pediatric patients with *PIK3CD* mutation-related immunodeficiency disease. Without posaconazole, the initial dosages of sirolimus were 0.07, 0.06, 0.05, and 0.04 mg/kg/day for body weights of 10–14, 14–25, 25–50, and 50–60 kg, respectively. With posaconazole, the initial dosages of sirolimus were 0.02 mg/kg/day for body weights of 10–60 kg.

## Data Availability

The original contributions presented in the study are included in the article/Supplementary material, and further inquiries can be directed to the corresponding authors.
