# Supplementary figures and images for: Initial sirolimus dosage recommendations for pediatric patients with PIK3CD mutation-related immunodeficiency disease
Source: Front Pharmacol. 2022 Sep 14;13:919487. doi: 10.3389/fphar.2022.919487 (PMC9515533; doi:10.3389/fphar.2022.919487)

**
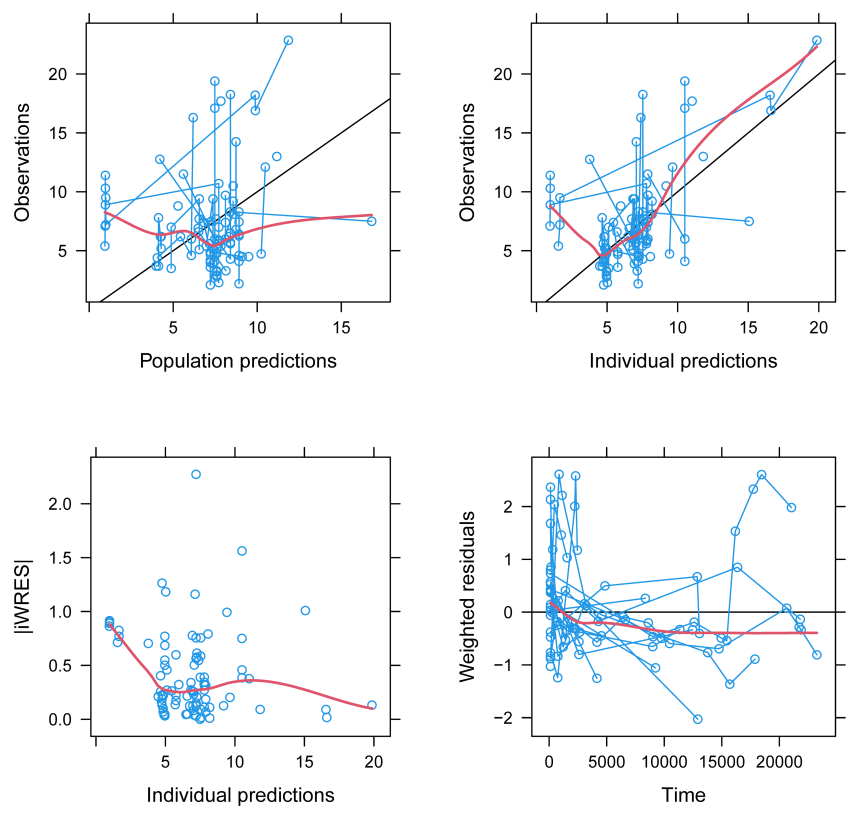
**

**goodness of fit plots (basic model)**

**
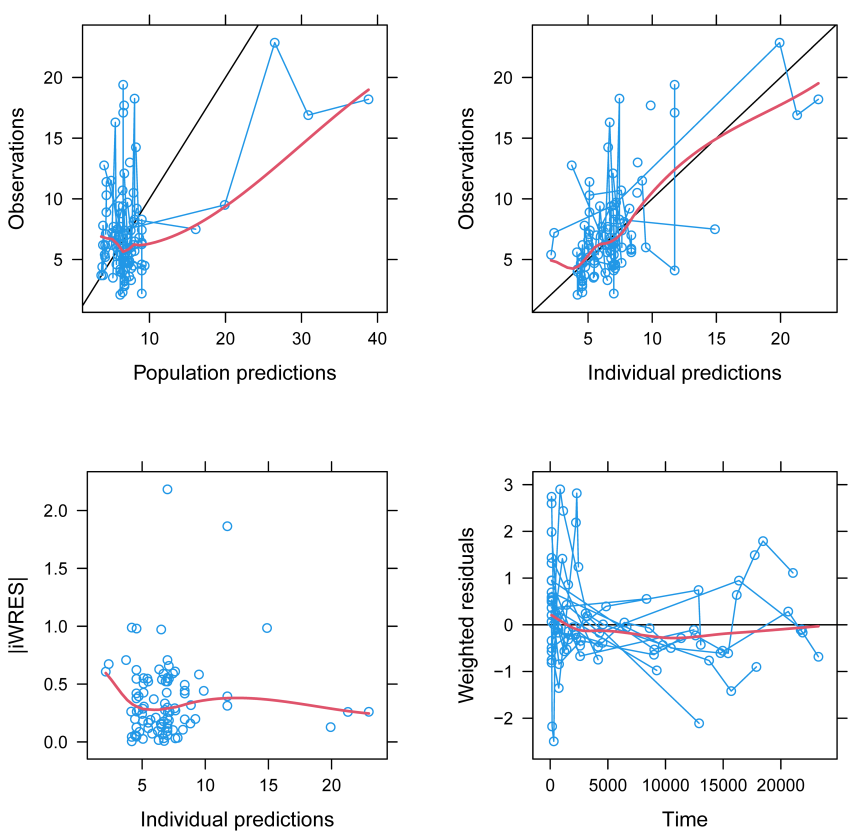
**

**goodness of fit plots (final model)**

Supplement: Supplementary file 1 [file Table1.DOCX]
